# Assessing the role of community involvement and capacity building in larviciding applications for malaria control in Africa: A scoping review

**DOI:** 10.1016/j.crpvbd.2025.100307

**Published:** 2025-08-14

**Authors:** GloriaSalome Shirima, Thiery Masserey, Hamenyimana Gervas, Nakul Chitnis, Samson Kiware, Silas Mirau

**Affiliations:** a Ifakara Health Institute, P.O. Box 53, Ifakara, Tanzania; b The Nelson Mandela African Institution of Science and Technology (NM-AIST), P.O. Box 447, Tengeru, Arusha, Tanzania; c University of Basel, Petersplatz 1, 4001, Basel, Switzerland; d Swiss Tropical and Public Health Institute, Kreuzstrasse 2, 4123, Allschwil, Switzerland

**Keywords:** Larviciding, Community engagement, Capacity building, Malaria control, Vector control

## Abstract

Larviciding offers a supplementary approach in malaria vector control, particularly when applied through community engagement and capacity building. A scoping review was performed to evaluate existing larviciding delivery mechanisms and their impacts on African malaria control. A scoping review was conducted following the PRISMA-ScR guidelines. The search strategy utilized Medical Subject Headings (MeSH) and free-text terms related to “malaria”, “larvicide”, “community engagement” and “mosquito control”. The databases PubMed, Scopus, and Embase were searched for relevant literature published until December 2024. Inclusion criteria focused on studies addressing community engagement in delivering larviciding within African settings. After applying inclusion and exclusion criteria, 32 papers were ultimately included in the analysis. The studies spanned 13 African countries, primarily in sub-Saharan regions, with findings indicating that larviciding significantly reduced mosquito density and, in some cases, malaria incidence. Community engagement strategies varied, with workshops and participatory meetings targeting various stakeholders to enhance awareness and ownership of larviciding programmes. Community engagement and capacity building were critical to successfully implementing larviciding programmes. While challenges, such as logistical barriers, lack of awareness, and financial constraints, persist, integrating technological innovations and strengthening monitoring systems can enhance the sustainability of these efforts.

## Highlights

• The scoping review evaluates existing larviciding delivery mechanisms in 32 studies, spanning over 13 African countries.
• Community engagement methods involved participatory planning meetings, sensitizations, use of local leaders and training.
• Methods enhanced local participation by fostering a sense of ownership and assuring the sustainability of the programme.
• Larviciding effectiveness is context-specific, influenced by community involvement and environment.

## Graphical abstract

**Figure:**
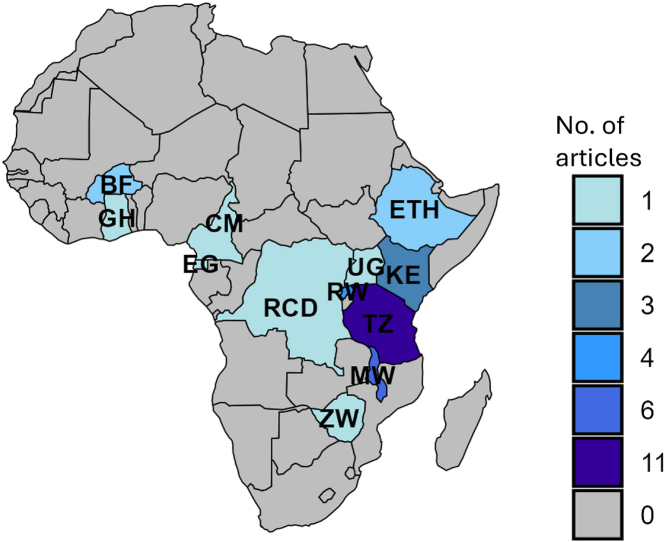

## 1 Introduction

Vector control is an essential strategy for combating vector-borne diseases, such as malaria. It was estimated that over 75% reduction in *Plasmodium falciparum* malaria cases is attributed to vector control interventions, mainly indoor residual spraying (IRS) and the use of insecticide-treated nets (ITNs) (Bhatt et al., 2015). The integration of IRS and ITNs has significantly reduced mosquito-human contact and controlled malaria vectors, hence reducing the burden (Protopopoff et al., 2015; Dulacha et al., 2022). However, the effectiveness of these tools is rapidly threatened by several factors, such as the emergence of insecticide resistance, and changes in vector composition, distribution, and behavior. These factors are increasingly challenging the cost-effectiveness, continuous success, and sustainability of these interventions.

Existing evidence indicates that larval source management (LSM) offers a sustainable, cost-effective, and environmentally friendly alternative tool for controlling mosquito vector populations (Worrall and Fillinger, 2011; Dambach et al., 2016). There are several mechanisms for the implementation of LSM for the control of mosquito vectors. These mechanisms include environmental modification, environmental manipulation and larviciding. While environmental manipulation and modification involve removing the mosquito breeding habitats permanently or temporarily (Martello et al., 2022), larviciding involves the treatment of the aquatic habitats without removing them. Larviciding is mostly applied in areas with few, fixed and findable mosquito breeding sites (Okumu et al., 2025). This targeted approach ensures control of the mosquito larval stage which significantly reduces adult mosquito density including vectors exhibiting outdoor behaviour that is not preventable with ITN and IRS (Moiroux et al., 2012; Dambach et al., 2016) and thereby lowers the biting rate of mosquitoes to humans, such as noted in studies highlighting changes in *Anopheles* spp. biting patterns following widespread ITN use (Moiroux et al., 2012; Dambach et al., 2016).

Larviciding in the sub-Saharan setting is commonly done using biolarvicides such as *Bacillus thuringiensis israelensis* (*Bti*), *B. sphaericus* (*Bs*), methoprene and pyriproxyfen. *Bti* is a naturally occurring bacterium that acts as a biological larvicide, targeting the larval stage of mosquitoes. *Bti* produces crystal proteins (Cry and Cyt toxins) that, when ingested by mosquito larvae, disrupt the gut lining, leading to lysis and death within hours (Schnepf et al., 1998; Lacey, 2007). *Bti* does not contribute to resistance development because its toxins act *via* multiple synergistic modes of action and are not persistent in the environment, hence offering a viable supplementary approach to the core methods (Rao et al., 1995; Fillinger and Lindsay, 2011; Imbahale et al., 2012; Kim et al., 2017). *Bs* is effective against various mosquito species and operates through similar mechanisms, producing toxins that disrupt the gut lining of larvae, leading to their mortality (Baumann et al., 1991; Lacey, 2007). Methoprene and pyriproxyfen are juvenile hormone analogs that prevent the transformation of pupae into adults (Mulla et al., 1986).

The choice of delivery for larvicides is critical as it directly or indirectly impacts the effectiveness and sustainability of these interventions against the transmission of *P. falciparum* (Imbahale et al., 2012). Community engagement and capacity building among local health workers, who are front liners in the implementation of this intervention, have emerged as key strategies to enhance the effectiveness and sustainability of larviciding. In this study, the term community is used inclusively to refer to a broad set of actors involved in larval source management (LSM) activities. This includes residents of the target geographical areas, local farmers and pastoralists whose agricultural and livestock practices influence mosquito breeding, grassroots groups and other local organizations, as well as local government authorities and programme implementers. Recognizing the diversity within and across these groups is critical, as each brings distinct perspectives, capacities, and forms of engagement that can shape the effectiveness and sustainability of community-based vector control initiatives. Community-based larviciding, where local communities are actively involved in the implementation of larviciding programmes, has shown promise as a supplementary approach on top of ITN and IRS for malaria control (McCann et al., 2021; Hakizimana et al., 2022). Engaging communities in vector control initiatives not only leverages local knowledge and resources but also fosters a sense of ownership, which is critical for the sustainability of these interventions (Maheu-Giroux and Castro, 2013; Mboera et al., 2014).

However, there is a need to explore how community engagement and capacity building can be involved in such programmes, as many mosquito habitats and breeding areas are created in the domestic environment as a result of agriculture and pastoralism activities (Mukabana et al., 2006; Lupenza et al., 2021; Kahamba et al., 2024). Importantly, community-based LSM approaches may offer a strategic advantage by enabling the identification and targeting of numerous, transient, and otherwise hard-to-locate breeding sites, thereby extending the reach of vector control efforts beyond the limited scope of fixed and easily identifiable habitats.

The application of larviciding in the community to ensure the control of malaria vectors with no proper delivery mechanism can likely not be cost-effective. The community engagement strategy will lower the cost of deploying this intervention. However, several challenges persist. This includes the labor-insensitive nature of work, time constraints, and insufficient financial resources, which hinder the effectiveness of larviciding programmes (Maheu-Giroux and Castro, 2013; Msugupakulya et al., 2024). On the other side of the capacity building, there are gaps in knowledge regarding mosquito habitats, insufficient monitoring, and limited financial resources, which complicate the implementation of larviciding (Kala et al., 2020; Feng et al., 2022). Addressing these challenges is essential for the development of larviciding strategies, ensuring they remain cost-effective and sustainable in the long term. A comprehensive evaluation of existing delivery mechanisms, gaps, challenges, and their effectiveness in various contexts is necessary to inform future vector control strategies (Tusting et al., 2013; Stuckey et al., 2014).

The successful implementation of effective LSM with optimal coverage requires community involvement at different stages of the design, planning, resource mobilization, and implementation. Therefore, understanding the impact of delivery mechanisms on the effectiveness of larviciding is essential in designing, planning, and implementing cost-effective and sustainable LSM. This scoping review explores the different types of community engagement and their role in delivering larviciding, by mapping out the existing studies that have used any of these mechanisms, by identifying scalable best practices, and lessons, and by reporting their impact on malaria vector control. The results of this study will ensure a robust application of larviciding in integrated malaria control strategies.

## 2 Materials and methods

This is a scoping review conducted to identify the different types of community involvement and capacity-building activities in larviciding applications and to evaluate the impacts of larviciding on malaria prevalence across various African settings. Automated artificial intelligence-based tools were integrated to facilitate data mining, including defining search terms, de-duplicating search results, and conducting title-and-abstract screening. This report adheres to the Preferred Reporting Items for Systematic Reviews and Meta-Analyses extension for Scoping Reviews (PRISMA-ScR) guidelines (Supplementary file 1).

### 2.1 Search strategy

To ensure a comprehensive synthesis of the available literature, an agreed-upon search and data extraction protocol was developed but not published, consisting of three electronic databases (PubMed, Scopus, and Embase). Searches on grey literature were not performed. Databases were searched for papers published until December 2024. No language or other restrictions were applied, and abstracts of publications in languages other than English were translated. Using a combination of medical subject headings (MeSHs) and free-text terms, search vocabulary related to “malaria”, “larvicide”, “Africa”, “community engagement”, “awareness” and “mosquito control” was employed. The terms used for the larvicide category included “biolarvicide”, “*Bacillus thuringiensis israelensis*”, and “larval source management”. Terms for community engagement included “participation”, while terms for awareness included “perception”. The Boolean operator “OR” was used for terms within word categories, “AND” between categories, combined with free text terms. An additional search of African-based databases was also conducted to identify any additional relevant regional literature.

### 2.2 Criteria for paper selection

The inclusion criteria for this review were based on the Population, Intervention, Comparison, and Outcome (PICO) framework as outlined in Table 1. These criteria were as follows: (i) studies conducting larviciding through community engagement; (ii) studies that measure the awareness or perception of the community on larviciding; (iii) studies that took place in an African setting either fields studies, randomized control trials (RCT) or cohort studies; and (iv) studies that applied larviciding to target malaria vectors.

**Table 1:** PICO criteria used to screen studies, indicating the characteristics of the included papers.

	Criteria
Population	Humans, any gender, any age: could be limited to one or more specific human population groups; OR Any life cycle stage of a vector causing malaria, all in African settings
Intervention/exposure	Exposed to a larviciding intervention, which is deployed through community engagement or awareness
Outcome	Prevalence, incidence, entomological indicators, mosquito density, and increase in awareness
Type of study	Cohort studies, field studies, randomized control trials

Exclusion criteria were: (i) studies outside the African continent; (ii) studies based on clinical case reports, laboratory, or semi-field settings on larviciding; (iii) the full text could not be obtained; (iv) pre-print version of the publication; (v) studies that target other non-malaria diseases or vectors; and (vi) articles published after December 2024.

### 2.3 Screening and extraction procedures

The Covidence software package (https://www.covidence.org/) was used to support record indexing, removal of duplicate records, title-and-abstract screening, full-text screening, and compilation of extracted data. After deduplication, screening of titles and abstracts of each record was performed. Non-English records were translated and reviewed in English. The Covidence title-and-abstract screening module incorporates a machine-learning algorithm that ranks papers that are yet to be screened according to their acceptance likelihood. Based on previous screening decisions, it pushes papers that are more likely to be accepted toward the top of the pile for all reviewers. This accelerates the screening process by removing the need to screen all search results; however, all papers were screened at this stage. Full texts were independently screened by two reviewers, with disagreements resolved through discussion.

Data was extracted from the full text of the selected papers that met the inclusion criteria. Data extraction was performed using a well-established data extraction form, and it was performed by two investigators independently. When there was a difference in the extracted information between the two investigators, they consulted a third investigator to sort it out. Extracted information included: the country where the study was done; the year of the study; how community engagement of capacity building was done; outcomes (entomological, epidemiological, community perceptions and awareness); recommendations and limitations or challenges from the study. All the extracted information is provided in Supplementary file 2.

### 2.4 Data analysis and synthesis

Data extracted from the articles was downloaded as an Excel file from the Covidence tool. R software (version 4.5) was used to visualize the countries in which studies were performed. The extracted data were analyzed and synthesized qualitatively to identify key engagement levels and strategies, community perceptions, entomological and epidemiological outcomes, gaps, challenges, and recommendations regarding the application of larviciding.

## 3 Results

**Fig. 1:**
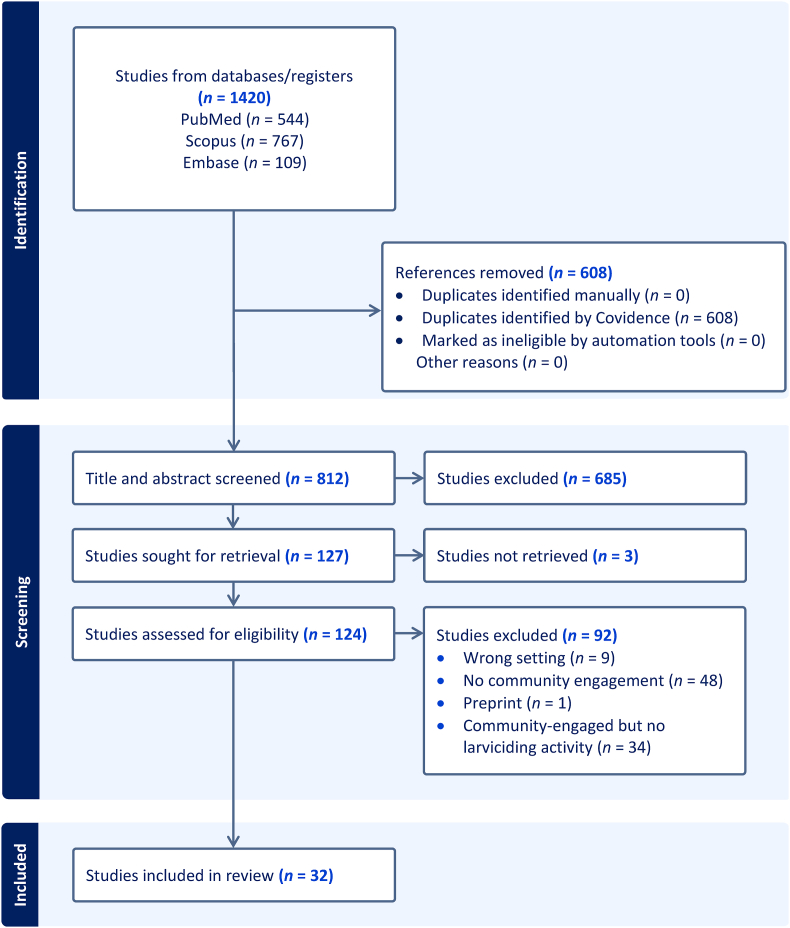
Flowchart illustrating the systematic review process, detailing the identification, screening, and inclusion of studies from various databases.

The database search yielded a total of 1420 papers, of which 608 (43%) were duplicates. After removing the duplicates, a total of 812 papers were available for title and abstract screening; 685 were excluded from the review at this stage due to not meeting the inclusion criteria of the study. A total of 124 papers were available for full screening, and 92 studies were excluded according to the exclusion and inclusion criteria of this study. Thus, a total of 32 papers qualified for the data extraction process and were included in this study (Fig. 1). Notably, six papers were removed from the study as they were not focusing on larviciding but other types of LSM. Activities such as drainage cleaning, removal of man-made habitat areas, and cleaning the environments were done using community engagement without inclusion of larviciding application and so excluded for data extraction (Yohannes et al., 2005; Vanek et al., 2006; Castro et al., 2009; Musoke et al., 2013; Ingabire et al., 2016; Djègbè et al., 2023).

**Fig. 2:**
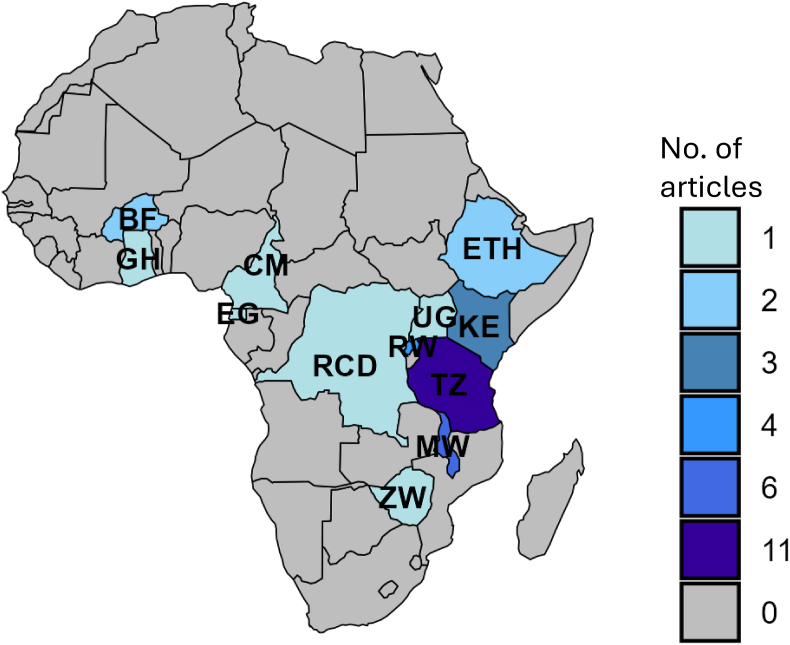
Map of Africa indicating the countries where papers included in the study were conducted and the number of papers obtained in each country.

Papers obtained on community engagement or capacity building on larviciding were conducted in 12 different countries across Africa (Fig. 2). All these countries are in sub-Saharan Africa, where malaria transmission is high. Articles found were published from 1947 to 2024. Of the accepted articles for review, 10 were focused on community engagement, while 22 addressed both community engagement and capacity building at the community level (Table 2). The larviciding targeted the major malaria vectors in the African setting, *Anopheles gambiae* and *Anopheles funestus*. These species were targeted in various aquatic habitats such as ponds, hoof prints, plantations, stagnant water, water pools, stagnant water in the swamps, and uncovered water-collecting instruments (Table 2).

**Table 2:** Overview of community-based malaria vector control studies. This table comprehensively analyses various studies focused on community engagement and capacity building in larviciding implementation. The data illustrate how different engagement strategies and capacity-building efforts influence larviciding outcomes and highlight the barriers faced in various settings, ultimately assessing their impact on malaria control.

Study area	Article title	Study setting	Targeted mosquito vector	Targeted habitat type	How community engagement or capacity building was done	Challenges outlined in the study	The impact observed on malaria control
Burkina Faso (Kossi Province)	A qualitative study of community perception and acceptance of biological larviciding for malaria mosquito control in rural Burkina Faso	Rural	*Anopheles* spp., *Aedes* spp., *Culex* spp.	Ponds, hoof prints, traditional brickworks, and partially wet rice fields	**Community engagement (passive):** The Community was engaged by being informed about the larviciding programme through personal observations, interactions with spray personnel, and local radio announcements, although radio was not a major source of information due to limited access. **Capacity building:** NA **Larviciding type:***Bti*	Not all water points were sprayed; there was a lack of awareness among some participants.	**Entomological outcome:** The majority of respondents reported relief from mosquito nuisance and malarial episodes.
Burkina Faso	Community acceptance of environmental larviciding against malaria with *Bacillus thuringiensis israelensis* in rural Burkina Faso - A knowledge, attitudes and practices study	Rural and semi-urban towns	*An*. *gambiae* (*s*.*l.*) and to a much smaller extent *An. funestus* and *An. nili*	–	**Capacity building**: The survey collected information on knowledge, attitudes, and practices regarding the intervention. It also sought to gather evidence on people’s trust, confidence, and willingness to contribute financially or with labor. **Larviciding type:***Bti*	The study acknowledges the hypothetical nature of willingness to pay questions and the potential for deviating behavior in actual payment scenarios.	**Entomological outcome:** More than 88.8% of the respondents confirmed the presence of mosquitoes at the time of the intervention, and 87.6% reported observing changes thereafter.
Cameroon	Knowledge, practices and perceptions of communities during a malaria larviciding randomized trial in the city of Yaoundé, Cameroon	Urban	*An*. *gambiae*, *An*. *coluzzii*, *An. funestus*	Mosquito breeding habitats in the intervention areas	**Community engagement:** In-depth focus group discussions were conducted to measure the perception and practices. Community participation was measured by the willingness of community members to allow access to their properties for larviciding activities, open their homes for mosquito collection, and propose members to work with the larviciding team. **Capacity building:** NA **Larviciding type:***Bti* and *Bs*	–	**Increased awareness:** Individuals in the intervention areas were 1.3 times more likely to know the mosquito breeding sites, with more testimonies on reduction in the number of cases and mosquito density due to larviciding application.
Equatorial Guinea	Evaluation of a multi-season, community-based larval source management program on Bioko Island, Equatorial Guinea	–	*Anopheles* spp.	–	**Community engagement:** The community was involved in both planning and implementation, with volunteers participating in the identification and treatment of breeding habitats. **Capacity building:** Training local volunteers to identify and treat mosquito breeding sites. **Larviciding type:***Bti*	A decrease in attendance and a lower amount of larvicide applied were noted.	**Entomological outcome**: During Phase I, 1033 breeding sites were identified with a 100% treatment coverage rate. Only 970 breeding sites were identified in Phase II, with a 75% treatment coverage rate, a significant decrease from Phase I (*P* < 0.001). Larvicide usage also decreased by 45% (95% CI: 32–59%, *P* = 0.003) between Phase I and Phase II.
Ethiopia	Community based integrated vector management for malaria control: Lessons from three years’ experience (2016–2018) in Botor-Tolay district, southwestern Ethiopia	Rural	*Anopheles* spp.	Various community-managed habitats	**Community engagement:** Community-wide events, meetings, and the establishment of community-based IVM working groups. School communities were also involved through co-curricular activities and IVM learning centers. **Capacity building:** Over 38,838 people received malaria education, and 612 individuals were trained in implementing IVM, including community members, student representatives, and health extension workers. **Larviciding type:***Bti*	–	**Entomological outcome:** Significantly fewer adult mosquitoes were collected in 2018 (0.37/house/trap-night) as compared to 2015 (0.73/house/trap-night) (*P* < 0.001). **Epidemiological outcome:** Malaria cases significantly declined in 2018 (*n* = 262) when compared to the record in 2015 (*n* = 1162) (*P* < 0.001).
Kenya	Are individuals willing to pay for community-based eco-friendly malaria vector control strategies? A case of mosquito larviciding using plant-based biopesticides in Kenya	Rural and urban	*Anopheles* spp.	Mosquito breeding habitats at the community level	**Capacity building:** The study used an auction experimental approach to engage the community and assess willingness to pay. Specific methods of community involvement in planning and implementation. **Larviciding type:***Bti*	Novelty of the biopesticide and the need for capacity building and communication to demonstrate its effectiveness.	**Increased awareness:** The study highlights a lack of awareness about larviciding, with only 23% of participants aware of *Bti* use, indicating a barrier to community participation. The study also reported that socioeconomic status, gender, marital status, age, household size, recent exposure to malaria sickness, income, and cost of treating malaria were significant factors affecting the use of the various control interventions.
Kenya	An exploratory survey of malaria prevalence and people’s knowledge, attitudes and practices of mosquito larval source management for malaria control in western Kenya	Peri-urban and rural highlands	*Anopheles* spp.	Plantations, stagnant water, water pools	**Capacity building:** Community participation was facilitated through surveys designed to explore perceptions of malaria risk, knowledge of mosquito breeding sites, and willingness to participate in control efforts. Developing community-based training programmes to raise awareness about man-made vector breeding sites and acceptable control methods.	–	**Awareness:** The study found that while LSM was frequently mentioned as a control tool, it was underutilized, with only 2 out of 90 respondents practising it. There was a gap between knowledge and action, with misconceptions about mosquito breeding sites prevalent.
Malawi	Community-based malaria control in southern Malawi: A description of experimental interventions of community workshops, house improvement and larval source management	Rural	–	Puddies, containers collecting rainwater, any stagnant sources that can support mosquito larvae	**Capacity building:** The community was involved through workshops, which served as a platform for education and collective action. These workshops included problem identification, planning, and evaluation. The training manual was developed, and three days of training were conducted, covering basics and practical aspects (surveying larval habitats).	–	**Increased awareness:** Increase the uptake of national malaria control recommendations and foster community mobilization. The impact of the LSM trainings on malaria control was assessed through coverage indicators, which were monitored throughout the project.
Malawi	Cost of community-led larval source management and house improvement for malaria control: A cost analysis within a cluster-randomized trial in a rural district in Malawi	Rural	–	–	**Community engagement:** Community members were actively involved in the planning and implementation of LSM. They conducted mapping of water bodies, draining, filling, and spraying activities. **Capacity building:** Workshops focusing on malaria-related topics were conducted regularly. **Larviciding type:***Bti*	–	The ‘epicentre approach’ was used to support communities in creating sustainable solutions, emphasizing sustainable malaria control as part of socio-economic development.
Malawi	Community factors affecting participation in larval source management for malaria control in Chikwawa District, southern Malawi	Rural	–	–	**Community engagement:** The community was actively involved in planning and implementing LSM activities. Local leaders played a crucial role in motivating. **Capacity building:** was facilitated through workshops, sensitization meetings, and the involvement of local leaders. These methods aimed to educate the community about malaria control and the safety of using microbial larvicides like *Bti*. Participation, and workshops were conducted to enhance knowledge about malaria and its control measures. **Larviciding type:***Bti*	The labor-intensive nature of LSM activities, time demands, lack of financial incentives, and initial concerns about the safety of *Bti*. These factors contributed to varying levels of participation among community members.	**Increased awareness:** Widespread knowledge of malaria as a health problem, its mode of transmission, mosquito larval habitats, and mosquito control was recorded. High awareness of an association between the creation of larval habitats and malaria transmission was reported. Perception of LSM as a tool for malaria control was high. The use of a microbial larvicide as a form of LSM was perceived as both safe and effective.
Malawi	Community participation in habitat management and larviciding for the control of malaria vectors in southern Malawi	Rural	*Anopheles* spp.	–	**Capacity building**: The community was involved through the formation of LSM committees, which coordinated activities following specialized training. Training covered topics such as mosquito biology, larval habitats, and the use of *Bti* for larviciding. Skills developed included recognizing mosquito larvae, operating spraying machines, and preparing *Bti*-water mixtures. **Larviciding type:***Bti*	Conflicting interests, such as the importance of waterbodies for livelihoods, were barriers to removing potential mosquito habitats. Less participation in habitat draining and filling activities was noted compared to other activities.	**Entomological outcome:** Reduced anopheline larval densities in post-spray sampling compared with pre-spray sampling (*P* < 0.0001). No differences were observed between anopheline larval densities during pre-spray sampling in LSM villages and those in non-LSM villages (*P* = 0.282). **Increased awareness:** The intervention increased community knowledge about malaria but did not significantly reduce anopheline larval densities due to low mosquito densities after other malaria control interventions. Success was measured through surveys and interviews, focusing on changes in knowledge and participation rates.
Malawi	The effect of community-driven larval source management and house improvement on malaria transmission when added to the standard malaria control strategies in Malawi: A cluster-randomized controlled trial	Rural	*Anopheles* spp.	Few, fixed, and findable habitats	**Community engagement:** Discussions between research staff, community leaders, and community members at the focal area and village levels. **Larviciding type:***Bti*	Low community buy-in in some villages was noted as a barrier, affecting the coverage and effectiveness of HI and LSM interventions.	**Entomological outcome:** The mean nightly EIR fell from 0.010 infectious bites per person (95% CI: 0.006–0.015) in the baseline year to 0.001 (95% CI: 0–0.003) in the last year of the trial.
Malawi	Household knowledge, perceptions and practices of mosquito larval source management for malaria prevention and control in Mwanza District, Malawi: A cross-sectional study	Rural	*An*. *gambiae*, *An*. *arabiensis*	–	**Community engagement:** Intensive community engagement workshops were facilitated by health animators throughout the MMP catchment area. **Capacity building:** These workshops focused on malaria-related topics and were conducted regularly.	–	**Increased awareness:** 5.2% of the study population knew about larviciding. Compared to respondents with primary education, those with secondary education were more likely, whereas those without education were less likely, to have high-level knowledge of mosquito LSM methods (AOR = 3.54, 95% CI: 1.45–8.63 and AOR = 0.38, 95% CI: 0.23–0.64, respectively)
Rwanda	Community-based biological control of malaria mosquitoes using *Bacillus thuringiensis* var. *israelensis* (*Bti*) in Rwanda: Community awareness, acceptance and participation	Rural	*Anopheles* spp., *Culex* spp., *Aedes* spp.	Stagnant water in the swamps, rice irrigation systems, and uncovered water-collecting instruments	**Community engagement:** The community was involved through local malaria action teams (CMATs) and rice cooperatives, which played a key role in planning and implementing larviciding activities. Pre-engagement meetings were held with rice farmers to facilitate community adoption of the intervention. **Capacity building:** Training sessions were conducted for spraying teams, covering malaria epidemiology, vector biology, larviciding protocols, and monitoring and evaluation. **Larviciding type:***Bti*	Concerns about the safety of *Bti* on rice growth and water use were initially reported, but were alleviated after implementation.	**Increased awareness:** The community perceived a reduction in mosquito density and nuisance biting, indicating a positive reception of the larviciding efforts.
Rwanda	Community-based control of malaria vectors using *Bacillus thuringiensis* var. *israelensis* (*Bti*) in Rwanda	Rural	*Anopheles* spp.	Rice fields, permanent and semi-permanent waterbodies	**Community engagement:** Rice farmers and leaders of community-based organizations were involved in the planning and implementation of LSM. **Capacity building:** Five-day training on *Bti* spraying was conducted for the CMATs. **Larviciding type:***Bti*	The effectiveness of larviciding was reduced when implemented by communities due to potentially less careful application and challenges in integrating it into daily farming practices.	**Entomological outcome:** In ES, the per-round reduction in *Anopheles* larval habitats was estimated at 49%. This reduction was less in CB (28%) and control (22%), although the per-round reduction in CB was still significantly higher than in control. Pupal production was almost completely prevented from round 5 (out of 10) onwards in both CB (average habitat occupancy 0.43%) and ES intervention arms (average habitat occupancy 0.27%), whereas pupal occupancy rates were on average 12.8% from round 5 onwards in the control.
Rwanda	Willingness to contribute to bio-larviciding in the fight against malaria: A contingent valuation study among rice farmers in Rwanda	Rural	–	Rice fields	**Community engagement:** The use of CMATs and rice farmers in the planning and application of larvicides. **Larviciding type:***Bti*	Challenges included low levels of acceptance by the local population due to perceived risks of bio-larvicides to other organisms and inadequate funding (willingness to pay).	**Increased awareness:** Financial contributions would be significantly different from zero and sufficient to carry a co-financing share of 15–25 per cent. A strong heterogeneity in mean WTP is revealed across cooperatives, in addition to variation among individual farmers, which needs to be anticipated when engaging farmer cooperatives in LSM.
Rwanda	Local resource mobilization for malaria vector control among Rwandan rice farmers: A pilot study into the role of community ownership	Rural	*An*. *gambiae* (*s.l.*), predominantly *An*. *arabiensis* and to a lesser extent *Anopheles gambiae* (*s.s.*)	Rice fields	**Community engagement:** The study involved rice farmers in two groups: one where farmers sprayed their fields under expert supervision and another where they organized the larviciding campaign themselves. **Larviciding type:***Bti*	Complexity of causality in community-led LSM and the interaction between agency and financial commitment.	**Increased awareness:** The high-agency (self-organised) group significantly outperforms the low-agency (expert-supervised) group in terms of maintaining a willingness to contribute financially.
Tanzania	Achieving high coverage of larval-stage mosquito surveillance: Challenges for a community-based mosquito control programme in urban Dar es Salaam, Tanzania	Urban	*Anopheles* spp., culicines	Natural habitats, agricultural artificial habitats, and non-agricultural artificial habitats	**Community engagement** through the employment of CORPs for routine larval surveillance. The engagement was operational, with CORPs following predefined schedules and reporting to ward supervisors.	Accessibility of habitats in urban settings is a major challenge due to fenced compounds and under-reporting of larvae, especially where larvicides were applied.	**Entomological outcome:** CORPs reported 66.2% of aquatic habitats, though low detection sensitivity for *Anopheles* larvae was noted. The study found that CORPs detected *Anopheles* larvae in only 12.6% of habitats that contained them, with a particularly low detection rate for late-stage *Anopheles* larvae (2.7%). This low sensitivity raises concerns about the effectiveness of larviciding in reducing mosquito density, as many habitats with potential breeding sites were not identified or treated.
Tanzania	Using pastoralist community knowledge to locate and treat dry-season mosquito breeding habitats with pyriproxyfen to control *Anopheles gambiae s.l.* and *Anopheles funestus s.l.* in rural Tanzania	Rural	*An*. *gambiae* (*s.l.*), *An*. *funestus* (*s.l*.)	Dry-season mosquito breeding habitats	**Community engagement:** The community was actively involved in planning and implementing larval source management (LSM) by identifying dry-season waterbodies used for livestock, which also served as mosquito breeding habitats. **Capacity building:** Community sensitization meetings were conducted using a participatory learning and action approach involving local government leaders, livestock field officers, community members (pastoralists and farmers), and the research team. **Larviciding type:***pyriproxyfen*	–	**Entomological outcome:** During baseline, the average proportion (±SD) of adult emergence was similar between the two clusters, with 0.89 ± 0.22 for the control cluster, and 0.93 ± 0.16 for the treatment cluster of breeding habitats. Following treatment with PPF, the average proportion (±SD) of adults that emerged in the treated breeding habitats was significantly lower (0.096 ± 0.22) compared to adults that emerged from larvae in the untreated habitats (0.99 ± 0.22) (*P* < 0.0001). Of all emerged adults, approximately 94% were *An. gambiae* (*s.l.*) and the remaining 6% were *An. funestus* (*s.l*.).
Tanzania	Rice farmers’ perceptions and acceptability in the use of a combination of biolarvicide (*Bacillus thuringiensis* var. *israeliensis*) and fertilizer application for malaria control and to increase rice productivity in a rural district of central Tanzania	Rural	–	Rice farms	**Capacity building**: Farmers were trained on mixing the fertilizer and *Bti* under supervision; they applied this to their farms, thus increasing their **personal engagement**. Semi-structured interviews and focus group discussions (FGDs) with rice farmers to gather their knowledge, attitudes, and perceptions regarding the intervention were performed. **Larviciding type:***Bti*	Initial fears about the safety of biolarvicides to health and paddy plants were reported, but these concerns were alleviated through participation and information dissemination.	**Increased awareness:** The intervention led to a perceived reduction in mosquito density and malaria risk, with high community acceptability and willingness to pay for the programme. Before the implementation of the intervention, working in the paddy farms was associated with mosquito bites, which interfered with their working hours.
Tanzania	Microbial larvicide application by a large-scale, community-based program reduces malaria infection prevalence in urban Dar es Salaam, Tanzania	Urban	*An*. *gambiae*	Urban breeding sites	**Community engagement:** The study was part of a larger community-based programme, the Urban Malaria Control Programme (UMCP), which involved residents in various activities, including mapping and surveillance of mosquito breeding sites, monitoring adult mosquito densities, and conducting household surveys. This community-based approach ensured that the programme was rooted in local contexts and benefited from community insights and participation. **Larviciding type:***Bti*	Operational challenges	**Entomological outcome:** Reduced crude annual transmission estimates (Relative EIR = 0.683 (95% CI: 0.491–0.952, *P* = 0.024) but programme effectiveness peaked between July and September (Relative EIR = 0.354 (95% CI: 0.193–0.650, *P* = 0.001) when 45% (9/20) of directly observed transmission events occurred. **Epidemiological outcome**: Larviciding reduced malaria infection risk among children ≤ 5 years of age (OR = 0.284 (95% CI: 0.101–0.801, *P =* 0.017) and protected at least as good as personal use of an insecticide treated net (OR = 0.764 (95% CI: 0.614–0.951, *P* = 0.016).
Tanzania	Impact of community-based larviciding on the prevalence of malaria infection in Dar es Salaam, Tanzania	Urban	*Anopheles* spp.	Various urban habitats	**Community engagement:** The community was involved through the employment of CORPs, who were responsible for routine mosquito control and surveillance. This approach ensured local involvement and ownership of the intervention. **Larviciding type:***Bti* and *Bs*	The start of larviciding phases did not always coincide with survey rounds due to operational issues, which could have affected the observed differences in malaria prevalence. The lack of reliable temporal information on ACT use and potential confounding factors due to seasonality in malaria transmission.	**Epidemiological outcome:** The study found a significant reduction in malaria prevalence over the survey rounds. The prevalence decreased from 20.8% in the first round to 1.7% in the last survey round.
Tanzania	Process evaluation of a community-based microbial larviciding intervention for malaria control in rural Tanzania	Rural	*An*. *gambiae*, *Culex* spp.	Ponds, rice paddies, brick pits, and temporary sites from rainfall	**Community engagement:** Community members were directly involved in the planning and implementation as larviciding staff. They were responsible for identifying breeding habitats and applying larvicide. **Capacity building:** Community members were recruited and trained by local leaders, entomologists, and a larvicide specialist. The training involved identifying mosquito species and applying larvicide. Larviciding type: *Bti*	Insufficient training, protocol adaptation, environmental and resource issues, and human error were identified as limitations affecting the intervention’s success.	**Entomological outcome**: The intervention was successful in terms of reach, as staff applied microbial larvicide at 80% of identified mosquito breeding habitats. However, the dosage of larvicide applied was sufficient to ensure larval elimination at only 26% of sites, which does not meet the standard set for intervention fidelity.
Tanzania	Community knowledge and acceptance of larviciding for malaria control in a rural district of east-central Tanzania	Rural	*An*. *gambiae* (*s.l.*), *An*. *funestus*	Rice paddies, ponds, puddles, roadside canals and ditches, streams, and temporary wetlands	**Community engagement:** The community was involved through structured surveys and discussions, which sought to understand their knowledge, attitudes, and perceptions regarding larviciding. The study also assessed willingness to contribute financially to the programme.	–	**Increased awareness:** Most survey respondents trusted in the safety (73.1%) and efficacy of larviciding, both for mosquito control (92.3%) and to reduce malaria infection risk (91.9%).
Tanzania	Addressing key gaps in implementation of mosquito larviciding to accelerate malaria vector control in southern Tanzania: Results of a stakeholder engagement process in local district councils	Rural and urban	*Anopheles* spp.	Freshwaters, standing waters, pit latrines, trash pits, septic pits, used tires, long grass, and bushes	**Capacity building:** Community engagement involved surveys and key informant interviews to assess awareness and perceptions regarding larviciding. Community sensitization was primarily conducted by ward health officers with assistance from community health workers (CHWs).	(i) Insufficient knowledge for identifying relevant aquatic habitats of malaria vectors and applying larvicides; (ii) Inadequate monitoring of programme effectiveness; (iii) Limited financing; and (iv) Lack of personal protective equipment.	–
Tanzania	A toolbox for operational mosquito larval control: Preliminary results and early lessons from the Urban Malaria Control Programme in Dar es Salaam, Tanzania	Urban	*Anopheles* spp.	Open to the sun and closed to the sun	**Community engagement:** The involvement of community-owned resource persons (CORPs) in mosquito control and surveillance was a practical and affordable strategy. This approach allowed for timely operational responses to changing ecological and programmatic conditions, which were previously unthinkable at this scale. **Larviciding type:***Bti*	Insufficient coverage by CORPs and poor training, support, and supervision at the early stages of the programme. These challenges underscored the need for improved training and support systems.	**Entomological outcome:** The programme reduced malaria transmission by the primary vector, *Anopheles gambiae* (*s.l.*), by 31% in the intervention’s first year. **Increased awareness:** The community engagement efforts improved standards of larval surveillance and management.
Tanzania	Institutional evolution of a community-based programme for malaria control through larval source management in Dar es Salaam, United Republic of Tanzania	Urban and peri-urban	*Anopheles* spp.	–	**Community engagement:** The programme involved community members in the planning and implementation of LSM activities, such as mapping and larvicide application, and engaged community members directly in these activities.	Local communities often had a limited understanding of the relationship between malaria, mosquito species, and habitats.	**Epidemiological outcome:** The programme effectively reduced malaria prevalence by over 70% at a cost of < $1 per person protected per year, comparing very favourably with gold standard interventions, such as LLINs and IRS in the early roll-out phase, demonstrating the success of community engagement efforts.
Tanzania	Biolarviciding implementation in southern Tanzania: Scalability opportunities and challenges	Urban and rural	–	–	**Community engagement** was attempted through meetings, but there was a lack of direct involvement and sensitization. **Capacity building:** Community leaders were often left to educate the community despite their limited knowledge. **Larviciding type:***Bti* and *Bs*	The community had limited awareness and involvement in the biolarviciding efforts, leading to a perception of inadequate implementation.	The study found low implementation of biolarviciding intervention in 9 out of 12 (75%) surveyed councils.
SSA	Bacterial larvicides used for malaria vector control in sub-Saharan Africa: Review of their effectiveness and operational feasibility	–	–	–	**Community engagement:** Community participation in larvicide applications and willingness to pay for interventions indicate a level of community involvement. **Larviciding type:***Bti* and *Bs*	High implementation costs and scarcity of trained personnel as limitations.	**Increased awareness:** The findings indicated that, at low application rates, bacterial larvicide products based on *Bti* and/or *Bs* were effective in controlling malaria vectors. The larvicide interventions were found to be feasible, accepted by the general community, safe to the non-target organisms, and the costs compared fairly well with those of other vector control measures practiced in SSA.
Kenya and Ethiopia	Evaluating the impact of larviciding with *Bti* and community education and mobilization as supplementary integrated vector management interventions for malaria control in Kenya and Ethiopia	Rural	*An*. *arabiensis*, *An*. *merus*, *An*. *funestus*	Fish ponds and water accumulation in shallow ground pits from the brick-making activity, shallow ponds, drainage channels, and wells for domestic water use	**Community engagement:** Community members, referred to as mosquito scouts, were involved in the application of *Bti*. **Capacity building:** People were trained locally by project staff, indicating a participatory approach in the planning and implementation of larviciding. **Larviciding type:***Bti*	Spillover effects and intermingling of residents from different clusters, which could have affected the clarity of intervention impacts.	**Epidemiological outcome:** Malaria prevalence was significantly reduced by 50% when using LLINs coupled with application of *Bti*, community education, and mobilization. The two other sites did not reveal significant reduction in prevalence as a result of combining LLINs with any of the other supplementary interventions.
Uganda	Integrated approach to malaria prevention at the household level in rural communities in Uganda: Experiences from a pilot project	Rural	–	–	**Capacity building:** The establishment of demonstration households, training of CHWs, and community sensitization activities.	–	**Increased awareness:** There was a statistically significant positive change in specific malaria prevention practices; awareness of ways to avoid malaria rose from 68.4% to 91.7%.
–	Larval source management for malaria control: Prospects for new technologies and community involvement	–	Anopheline mosquitoes	–	**Community engagement:** The Farmer Field School, Open Space, and citizen science are designed to motivate and involve communities in malaria control efforts. **Larviciding type:***Bti*	Maintaining long-term community engagement, particularly in terms of acceptability and participation, which are critical for the success of control programmes.	**Increased awareness:** The study reported increased community awareness and support for LSM, with significant correlations found between reported mosquito nuisance, actual mosquito numbers, and malaria cases.

*Abbreviations*: CHW, community health workers; CMATs, community-based malaria action teams; CORPs, community-owned resource persons; LSM, larval source management; *Bs*, *Bacillus sphaericus*; *Bti*, *Bacillus thuringiensis israelensis*; LLINs, long-lasting insecticidal nets; SSA, sub-Saharan Africa; IRS, indoor residual spraying; ACT, artemisinin-based combination therapy.

### 3.1 Larviciding through community engagement

A total of 32 papers were analyzed to assess the community engagement and involvement in the deployment of larviciding intervention. Key aspects examined were methods of community engagement, levels of community participation in planning and implementation, strategies used to increase engagement, and barriers to participation. Data extracted from these papers revealed a variety of approaches to community engagement among the studies.

#### 3.1.1 Methods of community engagement

Different studies used different methods to ensure community engagement. Common strategies included workshops (5/32 studies, 16%), focus group discussions (FGDs) (5/32 studies, 16%), and participatory planning meetings (10/32 studies, 31%), outlined in Table 2. Three papers indicated establishing community-based malaria action teams (CMATs) as platforms for engaging community members in planning, implementing and monitoring larviciding activities in the community (Ingabire et al., 2017; Koenraadt, 2021). Other studies used mosquito scouts, students and military personnel (Asale et al., 2019; Mutero et al., 2020), community health workers (CHW) (Mapua et al., 2021), Community-Owned Resource Persons (CORPs) (Fillinger et al., 2008; Chaki et al., 2009), rice farmers (Rulisa et al., 2021), and government local leaders (Ingabire et al., 2017; Gowelo et al., 2020) in different roles in the application of larviciding to the community. The selected CHWs, scouts and CORPs would come from the targeted community, ensuring they speak the same native language and are people known among the community. For the study conducted in Kenya and Ethiopia (Mutero et al., 2020), community members, referred to as mosquito scouts, were involved in the application of *Bti*. They were trained locally by the project staff, indicating a participatory approach in the planning and implementation of larviciding. This strategy is likely to increase their engagement and ownership of the intervention.

#### 3.1.2 Level of community participation in planning and implementation

The studies reported active participation of the community members in planning and implementing larviciding activity. Two levels were observed: first, a high level of participation, and second, an operational level of participation. High-level participation involved the community in both the planning and implementation phases. In 10 (31%) studies, the community itself took a significant role in both the planning and execution of larviciding intervention, contributing to activities such as identifying mosquito breeding habitats and applying larvicides indicated in Table 2. In Ethiopia, a study observed that community members who were scouts were first involved in planning, trained and tasked in identifying breeding sites and applying every other week 3 granules/hoofprint to 125–500 g *Bti* per hectare (Asale et al., 2019). This involvement leads to higher ownership and effectiveness levels. In 4 studies (13%), community engagement involved direct surveillance activities, such as mapping and monitoring mosquito breeding sites, often conducted by CHWs or local volunteers, as referred to in Table 2. In operational-level participation, communities will only be engaged in implementation tasks. In Tanzania, this kind of involvement stood as a barrier due to isolated implementation and inadequate community sensitization and planning. Two studies (6%) reported that community members made financial contributions to support larviciding campaigns, indicating a level of commitment and local ownership (Mboera et al., 2014; Diiro et al., 2020).

#### 3.1.3 Sustainability and increasing community engagement

Involvement of local leaders, local organizations, farmers and environmental practitioners, financial contributions approaches allowed boosting community and local people engagement. In 14 (44%) studies, the use of local community leaders and community-based organizations was strictly emphasized in planning and decision-making processes (Table 2). In 12 studies (38%), efforts were made to integrate larviciding activities with local agricultural or environmental management practices and align these efforts with community livelihoods (see Table 2). Educational programmes and training sessions were conducted for community members, mosquito scouts, and community health workers to ensure awareness and campaign for more participation in the activities involving larviciding implementations.

#### 3.1.4 Barriers to participation

The various barriers to community participation are identified differently in each paper, the most frequent ones include operational challenges, such as the labor-intensive nature of larviciding (Gowelo et al., 2020; Kamndaya et al., 2021), lack of financial incentives (Gowelo et al., 2020), and time constraints (Hakizimana et al., 2022). In 4 studies (11%), geographical barriers such as difficult terrain and heavy rains limited the effectiveness of manual larviciding. Concerns about the safety of larvicides like *Bti* were mentioned, although these were reduced after initial implementation and sensitization efforts (Mboera et al., 2014; Mazigo et al., 2019; Gowelo et al., 2020). Socio-political factors, including conflicts and war in Ethiopia, significantly disrupted community engagement and participation (Yohannes et al., 2005). Additionally, lack of knowledge or misconceptions about the connection between malaria transmission and mosquito breeding sites was noted as a barrier in 6 studies (19%) (Table 2).

### 3.2 Larviciding through capacity building, level of awareness, community perception and knowledge

Capacity-building efforts were a key component in the implementation of larviciding interventions across several studies. Various stakeholders, including CHWs, local volunteers, and government health officials, were enhanced with skills that will ensure the sustainability of larviciding activities.

#### 3.2.1 Training and skills development

Conducting training was a core component of capacity building. In some studies, before the community, CHW, CORPs, and scouts could participate in larviciding activities, training was conducted (Table 2) to equip necessary skills for identifying mosquito breeding sites, applying larvicides, and monitoring the effectiveness of interventions in mosquito control. Specific approaches like farmer field schools (FFS) were employed to educate local farmers on mosquito larval identification and control (Djègbè et al., 2023). In some papers, the mosquito scouts, or CORPs, underwent training to conduct routine larval surveillance and apply larvicides. For instance, Tanzania and Malawi focused on training CHWs to perform weekly surveys of mosquito breeding sites, allowing for more effective and sustainable intervention strategies (Mboera et al., 2014; Gowelo et al., 2020). Some studies (6/32, 19%), as outlined in Table 2, incorporated community sensitization meetings to assist in the dissemination of knowledge and information on malaria prevention and the safety of microbial larvicides like *Bti* before the community takes charge of applying the larvicides.

#### 3.2.2 Leadership development

Local leaders were equipped with skills and ensured their development through training. Local committees selected as CHW, CMATs, and scouts were equipped to take ownership of larviciding activities. Local leaders were empowered to coordinate community-wide larviciding activities, manage public awareness campaigns and ensure accountability within the community as outlined in Table 2. In Malawi, the leaders ensured to educate the community about malaria control and the safety of using microbial larvicides like *Bti* (Gowelo et al., 2023). In Rwanda, local CMATs were trained to take leadership and oversee the application of larvicides and coordinate with local health authorities (Ingabire et al., 2017).

#### 3.2.3 Perception and acceptance

Community acceptance and its perception on larviciding were shown to be dynamic across the studies; 14 (44%) studies were conducted to assess the level of acceptance, knowledge and perception of the community on this intervention (Table 2). In some papers, initial skepticism on the concerns about the safety of larvicide, particularly their effects on crops and water sources, was noted. However, this was resolved through sensitization efforts and involvement of community leaders, leading to an increase in acceptance of the intervention over time (Mboera et al., 2014; Ingabire et al., 2017; Gowelo et al., 2020).

In two studies from Tanzania and Burkina Faso, the use of local health workers influenced the level of acceptance because these community figures were trusted (Dambach et al., 2018; Berlin Rubin et al., 2020). The studies indicated that where communities were actively engaged in the planning and implementation of larviciding, there was a higher level of trust and acceptance. This was particularly true when local volunteers or community members were trained and included in the application of larvicides. Community perception and acceptance were observed to be improving over time with the ongoing engagement, education, and observed results in mosquito density reduction.

#### 3.2.4 Barriers to capacity building

Although capacity building is a central component of larvaciding, certain studies, as shown in Table 2, highlighted barriers. A study in Kenya highlighted a significant barrier to be the community’s lack of understanding of the connection between man-made habitats and malaria transmission, as well as the importance of controlling these habitats for their livelihood (Diiro et al., 2020). Initial fear about environmental impacts due to the application of larviciding was also a barrier observed in other studies.

### 3.3 Impacts on malaria control

Larviciding through community engagement and capacity building has shown positive impacts on malaria control, with several studies reporting on the reduction of malaria prevalence, malaria incidence, mosquito density, and entomological inoculation rate (EIR) (Geissbühler et al., 2009; Maheu-Giroux and Castro, 2013). A significant reduction of mosquito density was observed following application of larviciding with *Bti* and some areas noted a 49% drop in mosquito population (Hakizimana et al., 2022). Notably EIR was reported to drop from 0.010 infectious bites per person (95% CI: 0.006–0.015) in the baseline year to 0.001 in the last year (McCann et al., 2021). Malaria incidence and prevalence were mostly noted in studies that assessed the impact of the integrated vector management, where the core interventions are combined with larviciding application *via* community engagement, such as a study from Ethiopia (Asale et al., 2019) (Table 2). The impact on malaria control differed from place to place, and this was due to various factors and strategies that work well in one region may not be as effective in another due to these unique contextual factors.

## 4 Discussion

We reviewed the existing knowledge on engaging the community and building their capacity or awareness on larviciding application activities as an intervention for malaria control across Africa. It highlights that the success of larviciding activities, their effectiveness, and their sustainability for malaria control are improved through community involvement and building local capacity. Specifically, the reviewed studies used the CMATs, CHW, and selected members in the community to participate in the application of larviciding. This involvement was shown in high-level (active participation) from planning to implementation, or low-level participation (passive) of just implementation. The workshops and different sensitization programmes played a crucial role in increasing awareness of larviciding intervention and increasing participation in taking ownership of the programmes. Additionally, barriers such as socio-political instability, geographical constraints, and lack of incentives were consistently identified as significant challenges to community participation (Yohannes et al., 2005; Ingabire et al., 2016). Regarding the impact on malaria transmission, the studies generally indicate that larviciding has led to reductions in mosquito density, mosquito infection rate, and a decrease in malaria incidence or prevalence in some cases. However, the variability of results across regions suggests that the effectiveness of larviciding is context-specific, influenced by factors like the intensity of community involvement and the environmental characteristics of the targeted areas.

The strengths of this review lie in the availability of studies carried out in sub-Saharan Africa. Many of the studies selected for review had similarities, providing a comprehensive picture of how community engagement and capacity-building activities have been applied in larviciding contexts. The studies from Tanzania and Rwanda demonstrated participatory approaches influencing community perception and involvement in malaria control efforts (Chaki et al., 2009; Ingabire et al., 2017). The data extracted offer insights into how sensitization and awareness campaigns shaped local knowledge and acceptance of larviciding. The weaknesses seen in some studies are the hardship in quantifying the impact of the level of community participation or measuring the increase in the level of awareness/understanding of the community. Some studies gave a direct outcome to the reduction of incidence and prevalence, but this was possible only if no other interventions were performed in parallel. However, the diversity of the engagement mechanisms makes it difficult to compare studies or draw conclusions. The reporting standards and different outcome measures in all studies make it difficult to draw conclusions. Nevertheless, all have shown a positive impact of community engagement on malaria control, generalizing the importance of community engagement and capacity building within delivery mechanisms. While we focused on the three databases to identify relevant studies, it is important to acknowledge that there are numerous relevant studies in the qualitative social sciences (such as anthropology and human geography) that may be underrepresented in these databases. Consequently, our synthesis may not fully capture the broader understanding of community involvement in larval control.

The mechanism of using community participation and capacity building for larviciding has been seen in other regions apart from Africa, studies in Latin America and Southeast Asia. Despite their target to fight against malaria, Zika, or dengue, the application had several divergences and similarities. In Brazil, a study indicated the conduct of workshops, community engagements in the cleanup campaigns projects promoted and funded by both federal and state health departments, taking over the monitoring of the habitats and applying the larvicides, with limited initiative at the local community level (Caprara et al., 2015). Although the Brazilian centralised approach has facilitated rapid and wide-scale coverage of larvaciding, particularly in urban settings, it has also been criticised for failing to engage communities effectively to enable long-lasting sustainability (Maciel-de-Freitas and Valle, 2014). In Thailand, community members are engaged in the surveillance of standing water in their locations, as a contribution to the fight against *Aedes aegypti* (George et al., 2015).

The extent of technology integration in larviciding programmes is a major difference between African and non-African contexts. Technologies have been used in countries across Southeast Asia and Latin America, involving the use of GIS mapping to more accurately target hotspots where larvae are found; and drones for faster identification, mapping, and spraying local areas with larvicide (Wang et al., 2014). However, African nations have traditionally been limited by semi-automated techniques that, while effective in some ways, are time-consuming and difficult to scale up. For example, in Malawi, Tanzania and Kenya, larviciding was impeded by manual identification and treatment of mosquito breeding habitats that hindered the scaling up of their implementation (Derua et al., 2019; Mapua et al., 2021). In a study in Rwanda, drones were used in rice farms, leading to a high impact on the efficiency of the application of larviciding. In the future, studies in Africa might consider technological options that could be optimized for integration with larvicides using lessons from the use of drones and automated larval source management systems in other countries, such as Singapore and Malaysia (Chng et al., 2022). Digital tools for mapping and monitoring, however, have helped in the larviciding activities in Sao Tome and proven to be a cost-effective strategy during implementation (Vigodny et al., 2023).

Compared to other studies out of Africa, where community-based larviciding has shown significant reduction in the vector density and disease incidence, some strengths and weaknesses become evident. African studies have begun adopting similar participatory frameworks proven successful elsewhere, such as community workshops and sensitization meetings, improving local acceptance and participation rates (Chaki et al., 2014; Asale et al., 2019). However, African studies face challenges like a lack of financial resources for long-term community involvement and logistical difficulties posed by remote locations, unlike regions with stronger funding and infrastructure, where most of the activities are government-funded and a clear enough budget is set (Khatri et al., 2024). Additionally, while studies outside Africa often employed standardized evaluation methods, African studies frequently lacked consistency in monitoring and evaluation frameworks, as they are project-funded, making it difficult to compare intervention effectiveness across settings (Masvaure and Fish, 2022).

The significance of using community engagement and capacity building as delivery mechanisms has been shown in the findings of this review. Ensuring that national programmes adopt this mechanism of community participation in the planning, sensitization, and implementation will lead to efficient resource utilization and greater intervention sustainability. From a policy perspective, national malaria control/elimination programmes should consider scaling this intervention to the national level in appropriate settings, based on recommendations from different modelling studies and small-scale studies (Runge et al., 2021). Additionally, policies aimed at providing financial and logistical support to maintain community engagement over time are necessary to prevent the high dropout rates observed in some reviewed studies (García et al., 2022). Finally, policymakers must address structural barriers, such as inadequate transportation or security issues, that hinder the implementation of these programmes in more remote or conflict-affected areas.

Findings of this review open up several areas of future research and programme improvements. First, there is a need for more robust systems for monitoring and evaluation to ensure the intervention’s long-term impact in the fight against malaria in combination with other interventions. Many of the studies reviewed did little about the long-term sustainability of this intervention; hence, to evaluate whether the initial successes seen in community engagement can be maintained over time without continuous external support is crucial. However, sustaining community-based larviciding programmes in the current funding landscape is increasingly challenging. With recent reductions in funding from major global health donors such as the U.S. President’s Malaria Initiative and other bilateral agencies, financial sustainability is a growing concern. Future efforts must consider sustainable and diversified financing models, including domestic resource mobilization, integration with other public health initiatives, and engagement of local government and the private sector. National governments should allocate dedicated funding to support larviciding interventions. Additionally, countries in Africa with ALMA’s (African Leaders Malaria Alliance) End Malaria Councils should ensure that larviciding is prioritized within the resources mobilized through these platforms (ALMA, 2025). Secondly, adding to the community-based deployment, some innovative technological approaches like drones for habitat identification and applications, especially in areas that are hard to reach or large-scale habitats, can be explored. The role of technology, such as the use of mobile applications for reporting mosquito breeding sites or monitoring larviciding activities, is another area that has not been thoroughly explored in the African context and should be considered in future studies. African countries could benefit from adapting these technologies to local contexts, thereby overcoming logistical and financial challenges associated with traditional larviciding methods. Multisector collaboration, especially with sectors such as agriculture, environment, water, and urban planning, can help embed larviciding interventions into broader development programmes, increasing visibility and reach. This cross-sectoral integration can also unlock additional resources, foster community trust, and ensure larviciding aligns with local priorities and livelihood activities. Finally, there is a need for stronger integration of larviciding into national malaria control programmes. While community engagement and capacity building are critical, these efforts must be supported by national policies that ensure the sustainability and scalability of larviciding programmes. Moreover, there is a need for more interdisciplinary studies, including both experimental and qualitative approaches, to provide stronger insights into the causal pathways between community involvement, capacity-building initiatives, and reductions in malaria incidence, as well as to better understand the social dynamics driving engagement. Finally, it remains unclear how these community-based larviciding programmes interact with other malaria control interventions, such as ITNs and IRS, and whether there is potential for synergistic effects that could further reduce the burden of malaria. Existing mathematical modelling techniques assessing the impact of larviciding in combination with ITNs and/or IRS should explicitly quantify the added value of community-based larviciding strategies. Importantly, larviciding should only be implemented following proper environmental management and source reduction efforts to ensure its effectiveness and sustainability.

## 5 Conclusion

This review emphasizes the crucial role of community involvement and capacity building for the successful implementation of larviciding programmes aimed to control malaria in Africa. Our review reports numerous obstacles, like logistical barriers, financial constraints, and the labor-intensive nature of larviciding, that should be tackled to improve larviciding programmes. Experiences from studies outside Africa suggest that integrating technological innovations, enhancing monitoring systems, and adopting a multi-faceted approach to vector control can boost the effectiveness and sustainability of larviciding efforts. Future research should assess the long-term impact of larviciding on malaria transmission and explore ways to integrate these programmes into national malaria control strategies for maximum impact.

## Ethical approval

Not applicable.

## CRediT authorship contribution statement

**GloriaSalome Shirima:** Conceptualization, Methodology, Writing - original draft, Writing - review & editing. **Thiery Masserey:** Methodology, Data curation, Validation, Visualization, Writing - review & editing. **Hamenyimana Gervas:** Validation, Data curation, Writing - review & Editing. **Nakul Chitnis:** Conceptualization, Methodology, Supervision, Writing - review & editing. **Samson Kiware:** Conceptualization, Methodology, Supervision, Writing - review & editing. **Silas Mirau:** Conceptualization, Methodology, Supervision, Writing - review & editing.

## Statement on the use of AI-assisted technologies

While preparing this article, the authors used OpenAI’s to correct grammatical errors and improve readability. After using this tool, the authors reviewed and edited the content as needed. The authors take full responsibility for the content of the published article.

## Funding

This work was supported, in part, by the Gates Foundation [INV-070227, INV-025569] and the Swiss federal government’s Department of Education and Research. Under the grant conditions of the Foundation, a Creative Commons Attribution 4.0 Generic License has already been assigned to the Author Accepted Manuscript version that might arise from this submission.

## Declaration of competing interests

The authors declare that they have no known competing financial interests or personal relationships that could have appeared to influence the work reported in this paper.

## Data Availability

The data supporting the conclusions of this article are included within the article and its supplementary files.
